# Indications and Outcomes of Uterine Artery Embolization in Patients with Uterine Leiomyomas

**DOI:** 10.1155/2012/920831

**Published:** 2011-10-26

**Authors:** Hidenori Sasa, Tatsumi Kaji, Kenichi Furuya

**Affiliations:** ^1^Department of Obstetrics and Gynecology, National Defense Medical College, 3-2 Namiki, Tokorozawa, Saitama 359-8513, Japan; ^2^Department of Radiology, National Defense Medical College, 3-2 Namiki, Tokorozawa, Saitama 359-8513, Japan

## Abstract

*Objective*. To investigate the indication and limitations of uterine artery embolization (UAE), we retrospectively analyzed the case of patients with uterine leiomyomas who had undergone UAE. *Methods*. During the past 7 years, 25 patients with uterine leiomyomas had undergone UAE in our hospital. UAE was indicated in patients with menstrual disturbances such as hypermenorrhea or dysmenorrhea. The outcomes of this procedure for uterine leiomyomas were analyzed. *Results*. Improvement in the menstrual symptoms and/or reduction in the leiomyoma size after UAE were observed in 24 patients (96.0%). There was mean 67.9% reduction in the volume of leiomyomas at six months after UAE (*P* < 0.05). However, the symptoms recurred after UAE in 6 patients (24.0%), with multiple or intramural leiomyomas larger than 7 cm, which necessitated additional procedures. *Conclusion*. The indications and limitations of the UAE should be considered because of its noneffectiveness and/or recurrence in over 20% of patients with uterine leiomyomas.

## 1. Introduction

Several nonsurgical techniques are used for the treatment of patients with uterine leiomyomas, such as the recently developed magnetic resonance-guided focused ultrasound therapy. Uterine artery embolization (UAE) is an outpatient nonsurgical radiologic treatment of uterine leiomyomas. It is noninvasive and useful for women who refuse hysterectomy, as well as for those with a high surgical risk, including morbid obesity [1].

We retrospectively analyzed the cases of 25 patients with symptomatic uterine leiomyomas who had undergone UAE to clarify the indications and limitations of the procedure. 

## 2. Materials and Methods 

Data of 25 patients with uterine myomas who had undergone UAE between 2003 and 2009 in National Defense Medical College Hospital (Saitama, Japan) are listed in Table 1. The mean age of the patients was 41.7 years, and the standard deviation was 3.5 years. UAE was indicated in patients with menstrual disturbances such as hypermenorrhea or dysmenorrhea after obtaining their informed consent. UAE was also performed in 3 patients with severe complications and a high surgical risk. Their preexisting medical complications were as follows: diabetes mellitus and renal failure; chronic kidney disease and peritoneal dialysis; cerebral palsy and thrombosis. We detected 12 submucosal myomas and 13 intramural leiomyomas. The mean hemoglobin value before the UAE was 8.5 g/dL, and the standard deviation was 3.0 g/dL. 

The general indications and contraindications for UAE in patients with uterine leiomyomas in the Department of Radiology in our hospital are shown in Table 2. The UAE was performed under local anesthesia as follows: the uterine artery was catheterized through a right unilateral approach after puncturing the right femoral artery, which enabled embolization particles to be injected into a single or both the left and right uterine arteries. Gelfoam^®^ (Pfizer Inc., USA) was used as the embolization particle, and up to one sheet of that was broken into pieces for the unilateral embolization. 

The outcomes including complications of the procedure for uterine leiomyomas were analyzed. The overall reduction in the volume of leiomyoma was evaluated at six months after UAE by MRI or ultrasound. Hemoglobin values were also measured at two months after the procedure. We used the Student's *t*-test for statistical analysis. A two-tailed *P* < 0.05 was considered statistically significant.

## 3. Results

**Figure 1:**
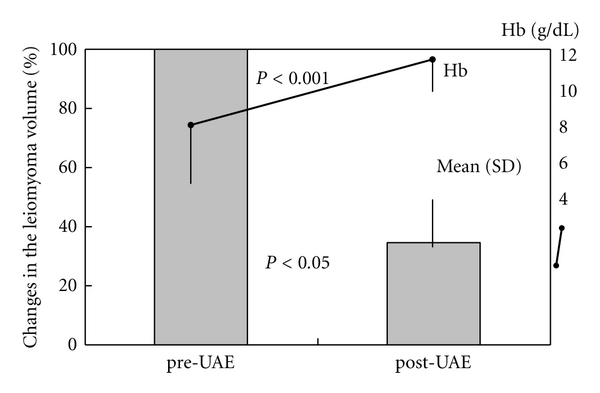
Changes in the leiomyoma volume (*n* = 17) and Hb values (*n* = 25) during course of the UAE treatment. UAE: uterine artery embolization; Hb: hemoglobin; SD: standard deviation.

The clinical outcomes of UAE in patients with uterine leiomyomas are shown in Table 3. They were followed up at least for 24 months. Improvement in the menstrual symptoms and/or reduction in the size of the leiomyoma were observed in 24 patients (96.0%). At six months after UAE, there was mean 67.9% reduction in the volume of leiomyomas in 17 patients, which was significant (Figure 1, *P* < 0.05). In 8 patients (32.0%), the submucosal leiomyomas were protruding and were thus removed after UAE. The anemia was significantly reduced at two months after UAE due to a decrease in the excessive uterine bleeding (Figure 1, *P* < 0.001). However, the symptoms recurred in 6 patients (24.0%), with multiple or intramural leiomyomas larger than 7 cm. The UAE was ineffective in 1 patient (4.0%) with a large leiomyoma. These patients required additional procedures such as repeat embolization (2 patients) and hysterectomy (3 patients). Although all the patients developed common complications such as abdominal pain, nausea, and fever, they exhibited immediate recovery without major complications. A patient revealed elevated liver enzymes and other patient complained of leg pain and recovered within several months. No patient went into menopause after UAE.

## 4. Discussion

UAE involves a short treatment using conscious sedation, an incision in the groin to access femoral vessels, and an overnight stay for pain control [2]. Our study showed that improvement in the menstrual symptoms and/or reduction in the size of the leiomyoma after the UAE were observed in most cases. Studies including randomized clinical trials in Europe document long-term symptom control for 1–5 years with improvement in bleeding, pain, and bulk symptoms [3, 4]. In other observational studies, UAE was followed by a significant reduction in uterine volume (35–60%), a decrease in excessive uterine bleeding in 90% of the women, a low rate of subsequent hysterectomy, and a high rate of sustained symptom control (up to 80%) 5 years after the procedure, as observed in our study [5].

However, persistent symptoms after UAE occur in approximately 20% of women who subsequently require other procedures such as repeat embolization, hysterectomy, or myomectomy [1]. In our study, the symptoms recurred in 24% of the patients who required repeat embolization and hysterectomy. Solitary leiomyomas measuring more than 10 cm or multiple fibroids with a uterine volume consistent with a 20-week or greater gestation are considered contraindications to UAE, due to its ineffectiveness on these leiomyomas [2]. UAE is particularly useful in women with a solitary submucosal leiomyoma smaller than 7 cm, and not those with multiple or intramural leiomyomas larger than 7 cm, as observed from another report and our results [6].

All the patients in our study developed common complications such as abdominal pain, nausea, and fever, followed by immediate recovery without major complications. One patient revealed elevated liver enzymes and another patient complained of leg pain temporarily, and no patient went into menopause after UAE. The ovarian impairment seen after UAE may be an asset for perimenopausal women [2]. However, UAE is of particular concern in women who wish to conceive. The resulting ovarian damage might not necessarily result in menopause but may impair future fertility. The safety of pregnancy after UAE remains unclear. The rate of a miscarriage, preterm delivery, postpartum hemorrhage, and placental abnormalities in the subsequent pregnancy after UAE were reported to be higher than that after a myomectomy [1]. UAE could be considered for infertile women who do not wish to undergo surgery or who are at poor surgical risk. They should be informed about the benefits and risks of the procedure including those of future fertility [1].

## 5. Conclusion

UAE is noninvasive and useful in women who refuse hysterectomy, as well as for those with a high surgical risk, including morbid obesity. It is particularly useful in women with a solitary submucosal leiomyoma smaller than 7 cm, but not in those with multiple or intramural leiomyomas larger than 7 cm. It is necessary to consider the indications and limitations of UAE and obtain prior informed consent in patients with uterine leiomyomas, who wish to undergo conservative managements, because this procedure is not effective in approximately 20% of these patients who subsequently required other procedures such as a hysterectomy. 

## Figures and Tables

**Table 1:** Patient characteristics (*n* = 25).

Age (y)	41.7 ± 3.5 (mean ± standard deviation)
Parity	0.96 ± 1.2
Indication of UAE	
Hypermenorrhea/anemia	20
Dysmenorrhea	5
Medical complications	3
DM · renal failure; CKD · PD; CP · thrombosis	
Type of uterine leiomyomas	
Submucosal	12
Intramural	13
Size (diameter, cm)	8.4 ± 5.3
Anemia (hemoglobin value, g/dL)	8.5 ± 3.0

UAE: uterine artery embolization; DM: diabetes mellitus; CKD: chronic kidney disease; PD: peritoneal dialysis; CP: cerebral palsy.

**Table 2:** General indications and contraindications of uterine artery embolization in patients with uterine leiomyomas.

Indications	
(1) Hypermenorrhea and/or dysmenorrhea	
(2) Absence of a malignant tumor in the uterus and lesion in the adnexa	
(3) Absence of a pelvic inflammatory disease	
(4) No childbearing	
Contraindications	
(1) No symptoms	
(2) Large leiomyoma (more than 10 cm in diameter) and/or multiple leiomyomas	
(3) Coexistence of a malignant tumor in the uterus or a lesion in the adnexa	
(4) Active pelvic inflammatory disease	
(5) Hormone therapy (required for 8–12 weeks after the therapy)	
(6) During pregnancy	
(7) Menopause	
(8) Hope of childbearing	
(9) Allergy to iodine	

Department of Radiology, National Defense Medical College Hospital, Japan.

**Table 3:** Clinical outcomes of UAE in patients with uterine leiomyomas (*n* = 25).

Improvement of symptoms and/or reduction in the leiomyoma size	24 (96.0%)
Reduction in the leiomyoma volume at 6 months after UAE (*n* = 17)	67.9 ± 16.3% (*P* < 0.05)
Removal of submucosal leiomyoma	8 (32.0%)
Improvement of anemia	
Hemoglobin value at 2 months after UAE (g/dL)	8.5 ± 3.0 →11.5 ± 1.5 (*P* < 0.001)
Prognosis	
No effect	1 (4.0%, a large myoma)
Hysterectomy	3 (12.0%)
Recurrence	6 (24.0%, repeat UAE 2)
Complications	
Abdominal pain, nausea, and fever	all the patients, short term
Elevated liver enzymes	1
Leg pain	1
Menopause	none

Mean ± standard deviation.
